# Condoms and condiments: compatibility and safety of personal lubricants and their use in Africa

**DOI:** 10.7448/IAS.16.1.18531

**Published:** 2013-07-09

**Authors:** Scott Geibel

**Affiliations:** Population Council, Lusaka, Zambia

**Keywords:** lubricants, condoms, HIV prevention, Africa

## Abstract

Previous research on the use of personal lubricants for sexual intercourse is limited and has primarily focused on condom compatibility and breakage, with only recent limited assessment of lubricant safety and possible epidemiologic implications. This article discusses the global evidence of lubricant compatibility with latex condoms and biological safety of lubricants, as well as documentation of lubricant use and current guidelines for HIV prevention programming in Africa. Data on lubricant compatibility with condoms are less available than commonly realized, and many lubricant products may not have been thoroughly tested for safety due to flexible regulatory environments. Recent laboratory and study findings from microbicides research also suggest that some water-based lubricants may have safety issues. Some African populations are using several types of lubricants, especially oil-based petroleum jellies, and receive little evidence-based guidance. More research is needed from the medical community to guide prevention programming.

## Introduction

Among the vast catalogue of African condom legends, this author’s favourite is a mythological experiment heard in Malawi 15 years ago. A young student reported that pouring *piri-piri* sauce – a popular spicy food sauce in Malawi – on a condom-sheathed penis would induce a burning sensation; hence proof that condoms have holes in them and cannot be trusted. This is symptomatic of most condom myths in Africa, which hypothesize inherent physical flaws in condoms themselves. But there is another hypothesis to the *piri-piri* experiment the student did not consider: Did the hot sauce itself weaken or damage the condom, therefore facilitating penetration of the latex barrier? Could it be possible that some myths in Africa result not of the *condom*, but of the *condiments* that are applied?

Table condiments aside, substances used during sexual intercourse can be applied for the purpose of lubricating the penis and the vagina or rectum in which it is inserted. This eases discomfort that may arise from dry friction during sex, and can increase pleasure or enjoyment. In Africa, however, most HIV prevention programmes focus little, if at all, on lubrication issues. Additionally, some marketing and packaging of government-distributed or socially marketed condoms do not strongly emphasize public advice on compatible lubricants.

Recent increased awareness of same-sex and anal sex practice in Africa has highlighted the lack of provision of personal lubricants [1,2]. Prevention programmes that provide lubricant supplies usually follow near-universally accepted recommendations that oil-based lubricants are to be avoided, and only water-based products should be used with condoms [3,4]. But has the potential epidemiologic impact of lubricant use in Africa – condom or no condom – been adequately assessed? Towards answering this question, it is worthwhile to review the global evidence regarding the effect of lubricants on condom structural integrity and the biological safety of lubricant use, as well as the prevalence of lubricant use and current guidance for prevention programming in Africa.

## Discussion

### Effect of lubricants on latex condoms: global evidence

Two ways to measure the effect of lubricants on latex condoms have been documented in the medical literature. One method is to apply lubricants in a laboratory setting and conduct precisely measured mechanical strength or burst tests. The other is to recruit actual condom users and measure lubrication use and condom breakages, either within a prospective cohort or retrospectively among a cross-sectional sample. Strength or burst testing is perhaps the easiest to measure and observe, as it is done in a controlled laboratory setting. Associating lubricant use with condom breakage in study populations is more challenging, since this relies on self-reported data and can be confounded by other factors such as incorrect use or slippage.

Condoms are usually tested according to standardized guidelines, such as ISO 4074:2002 “Natural latex rubber condoms – Requirements and test methods,” [5] and ASTM D3492 – 08 “Standard specification for rubber contraceptives (male condoms)” [6]. In 2011, the United States Food and Drug Administration (FDA) added ASTM D7661 – 10 “Standard test method for determining compatibility of personal lubricants with natural rubber latex condoms” to its list of recognized standards for use in pre-market product reviews [7,8]. It is not clear, though, to what extent previously marketed lubricant products, including those *not* marketed for sexual use, meet any of these standards.

The most-cited laboratory evidence describing condom breakage and oil-based substances is a 1989 paper published in *Contraception*, where the authors conducted burst testing with various substances, and concluded that mineral oil-based lubrications such as baby oil or body lotion had a significant degrading effect on latex [9]. A careful reading, however, reveals that Voeller *et al*. did not test petroleum jellies in this analysis, as is sometimes implied when this article is referenced. From the same year, an abstract from the 5th International AIDS Conference claimed that similar mechanical testing found petroleum jelly to have a significant weakening effect on latex condoms [10]. A 1999 study reported similar testing with vaginal application products, and found that products containing vegetable oil to weaken latex condoms [11]. A 2011 study reported that two commercial lubricants tested (one oil-based and one silicone-based) and mineral oil were found to decrease condom puncture strength, while all water-based products tested had a significant strengthening effect. A search of the indexed medical literature reveals limited laboratory-based evidence to test condom compatibility of petroleum jelly and silicone-based lubricants.

Numerous studies have examined condom breakage and slippage during vaginal intercourse among study populations. Breakage rates of less than 4% in the United States [12,13], 4% in Kenya and Mali and 13.3% in Ghana have been documented [14]. Another study also found breakage rates of 12.9% in Barbados, 10.1% in St. Lucia and 6.7% in the United States to be uncorrelated with breakage rates of same-lot condoms in the laboratory tests, and concluded the breakages were more likely due to incorrect use or other factors [15]. A study of men in Mexico, the Philippines and the Dominican Republic also concluded that reasons for breakage were complex and usually associated with historical breakage among individuals [16].

A 1993 assessment of men who have sex with men (MSM) in the Netherlands documented much lower condom failure rates among users of water-based lubricants (1.7%), than those who used oil-based lubricants (10.3%) or saliva/no lubricant (5.7%) [17]. Other results in the literature have been mixed. In 1999, a study of MSM in the United States also found lubricant use to have a protective effect against condom breakage, but the types of lubricant used by respondents were not described [18]. A 1996 study also reported that users of water-based lubricants reported significantly lower condom failure rates, yet at the same time showed no significant association between breakage and use of oil-based lubricants [19]. Some other studies have also failed to demonstrate significantly lower breakage rates due to oil-based lubricants [20,21]. One study of female sex workers (FSW) in Europe reported an association between use of oil-based lubricants and HIV positivity, but the authors did not relate this finding to possible breakage [22]. More recent studies, however, have reported users of oil-based lubricants were significantly more likely to have experienced condom breakage, including among African–American men in the United States [23] and male sex workers in Mombasa, Kenya [24].

### Safety of lubricant products: regulation and emerging global evidence

There is a lack of documentation of the human safety of personal lubricants, which may have resulted from a flexible regulatory environment. Many developing countries lack the capacity to monitor and review the safety of pharmaceutical products, and often rely on guidance from more stringent regulatory bodies such as the FDA or the European Medicines Agency [25]. This influence also extends to international HIV programming, where the President’s Emergency Plan for AIDS Relief’s (PEPFAR) and other US-based initiatives purchase and/or distribute these products. For example, the FDA approves the PEPFAR purchase of specific anti-retroviral drugs for use in cooperating countries [26].

The FDA has usually classified some personal lubricants as “medical devices” since 1976, depending on marketing or claim of the product itself. Under the 1976 FDA regulation 21 CFR 880.6375, a lubricant is considered a “medical device” when “intended for medical purposes that is used to lubricate a body orifice to facilitate entry of a diagnostic or therapeutic device.” Personal lubricant products that claim to “moisturize” or “cleanse” have often been considered as a “cosmetic.” In 2003, the FDA announced a safety and efficacy review, and clarified how moisturizer/lubricant product claims to decrease pain, enhance sexual pleasure or contain spermicide would be categorized. Such product statements would be considered to be “drug” claims since they are related to easing discomfort or alleviating a condition (“mitigation or treatment of disease”). Furthermore, the FDA announcement stated they would *not* consider lubricants/moisturizers to be “cosmetic claims because they do not relate to ‘cleansing, beautifying, promoting attractiveness, or altering the appearance’” [27].

In response to the FDA review, some lubricant manufacturers and their representatives have opposed the FDA’s assertions, arguing that the “intended use” of lubricants is “cosmetic claims,” as their products aim to “cleanse” and not “treat disease.” Arguments included that the purpose of the lubricant products is not to alter body structure, and that the enhancement of sexual pleasure can broadly be interpreted as “beautifying” [28–30]. While this is not a comprehensive review of responses to the FDA, the arguments for “cosmetic” classification may, or may not, be influenced by a possibility that having lubricants classified as “drugs” by the FDA could add further regulatory burden upon lubricant manufacturers to test for safety. In 2008, the FDA stated that further guidance on lubricants and vaginal moisturizers is forthcoming [31], but this guidance does not yet appear to be final or readily accessible.

In the meantime, the regulatory environment does not appear to always require lubricant manufacturers to rigorously confirm the vaginal and rectal safety of these products, especially if the product does not claim to be intended for sexual use. However, thanks to an emerging interest in the development of vaginal and rectal microbicides, new information is emerging.

Preliminary data suggest that some water-based lubricants – depending on their formulation – may be less safe than previously believed. One clinical study reported greater rectal epithelial damage when exposed to hyperosmolar water-based gels [32]. Recent studies have also evaluated *in vitro* and *ex vivo* impacts of some water- and silicone-based lubricants, and found that some caused greater epithelial damage or irritation to cervical or rectal tissue than others [33–36]. Among these results, one study found that some hyperosmolar lubricants exposed to an *in vitro* epithelial model caused reduction of epithelial integrity, and four lubricants showed increased HIV replication *in vitro* [35]. Another recent study of some water-based lubricants found similar associations with epithelial damage *in vitro*, but with no increase in HIV replication [36]. These studies provide preliminary insights, but have not yet demonstrated definitive epidemiologic impacts on human populations.

Evidence from behavioural data remains limited, as lubricant use has not often been assessed as HIV or STI risk factors in previous studies. One recent study, however, has found that rectal STI prevalence among lubricant-using men and women were significantly higher than non-lubricant users [37]. Combined with the recent laboratory evidence, this may be the cause for concern and more data are needed.

### Use of lubricants in Africa

Both practices of dry sex and use of lubricants for the increase of pleasure, decrease of pain or replacement/removal of vaginal secretions have been documented in Africa; as has vaginal hygiene or washing practices – especially among sex workers [38]. General personal lubricant use, however, is rarely investigated in population-based surveys. It is only more recently that some studies have assessed lubricant usage, albeit among more key populations at risk such as MSM and FSW.

A 2004 study of MSM in Nairobi reported that Vaseline^®^ or petroleum jelly was used by 84% of respondents, baby oil or body lotion by 26% and water-based products by 41% [39]. Other MSM studies in Africa have also reported high levels of petroleum jelly use and sometimes other “condiments,” such as butter/margarine, yoghurt, shea butter, and cooking oils [40–42].

Information on lubricant use for heterosexual vaginal intercourse in Africa is less available, though limited evidence suggests that oil-based lubricants may often be used in heterosexual encounters. Separate studies of FSW in Nairobi, Kenya, have documented petroleum jelly use by 20–40% of participants [43,44]. Additionally, male sex workers in Mombasa reported using such lubricants as petroleum jelly, baby oil, lotions, vegetable oil, and coconut oil with both male *and* female sexual partners for both anal and vaginal sex (Population Council, unpublished data).

Petroleum jellies may be a lubricant used for sex in many African countries, regardless of types of sexual intercourse or sexualities. These products, such as Vaseline^®^, are widely available and can be purchased everywhere from a city supermarket to remote rural kiosks. They are also relatively inexpensive compared to water-based pharmaceutical products such as KY Jelly^®^, which are usually only accessible in supermarkets and pharmacies. These factors may make petroleum jellies more attractive options in developing or poorer areas in Africa [45].

### Current guidance for lubricant procurement and prevention programming in Africa

Confidence in the condom-compatibility of water-based lubrication appears to be universal, and past guidance has strongly recommended their use [3,4]. However, the emerging evidence of possible correlations between use of water-based lubricants and HIV/STI transmission (especially rectal) has influenced some updated guidance. The PEPFAR guidance for combination prevention for MSM [46] recommends only procuring lubricants deemed safer by evidence presented at the Microbicides 2010 conference [36,37]. The PEPFAR guidance, however, does not specifically summarize which lubricant products are preferable.

In 2011, the World Health Organization (WHO), in collaboration with United Nations Population Fund and Family Health International, published an advisory document which reconfirms that oil-based lubricants should be avoided, and provides more clarity on the procurement of safer water-based lubricants. A list of household products are listed as damaging to latex including: baby oil, burn ointment, dairy butter, palm or coconut oil, cooking oil, fish oil, mineral oil, suntan oil, haemorrhoid cream, petroleum jelly and body/hand lotions [47]. This WHO list, however, references a behavioural study in Jamaica [48] which actually does not specifically mention, or scientifically test the condom compatibility of most of these “condiments,” as is implied.

Based on the evidence documenting epithelial or mucosal damage and/or irritation [32,34,35], the WHO advisory recommends a systematic review of lubricant safety, and provides interim recommendations for procurement agencies to avoid products with high osmolality and products containing polyquaternary compounds. Additional pH specifications for lubricants intended for vaginal or anal sex are provided [47]. The WHO document relists some commercial lubricants with their osmolality and pH from one of the recent studies [35], but – as with the PEPFAR guidance – stops short of declaring specific products as “safe” for procurement.

Based on this guidance, it seems that programmers are currently expected to independently research the formulation or ingredients of lubricant products before procurement. Additionally, in Africa water-based lubricants remain costly, and prevention programmes also remain unable to advise on the safety and condom compatibility of some inexpensive “condiments” reportedly used by MSM and FSW in Africa – including butter, yoghurt and coconut oil. In response, some organizations, most notably the International Rectal Microbicides Advocates (IRMA), are monitoring these research developments, distributing summaries and fact sheets of lubricant safety, and advocating for greater research resources on lubricant safety and use both in Africa and worldwide [49].

## Conclusions

The literature and evidence of lubricant condom compatibility and safety is not as thoroughly assessed and/or documented as commonly believed, and our knowledge is currently evolving and improving. This includes emerging awareness that Africans are using lubricants that may or may not be compatible with condoms, and that some Africans are engaging in anal sex, which requires more access to water-based lubricants. Additionally, further confirmation is needed on the safety and procurement criteria for some water- and silicone-based lubricants. Thus, the potential epidemiologic impact of lubricant use is under-assessed, and more attention must be given.

First, the current global regulatory environment for lubricants may not always require rigorous safety testing and assessment, especially if a product is not marketed for sexual use. Whether lubricants or vaginal moisturizers should be classified as “drugs” or “cosmetics” and tested for safety accordingly is currently under debate, while Africans continue to use lubricants based on intuition, availability and affordability – sometimes with no knowledge of the current public health guidance. While the recent information on vaginal and rectal safety testing from microbicides research has been helpful, further guidance from the FDA and more research is needed to resolve outstanding safety concerns. This will help assure HIV prevention programmers and lubricant users that they are distributing or using lubricants which are safe, and do not put Africans at increased risk of HIV or STIs.

Second, a thorough and modern review of lubricants and their impact on latex condom integrity is needed, including assessment of some Africa-specific substances. Confirmation of latex-degrading effects from the laboratory and breakage rates from behavioural data may be modelled with the existing data on the probabilities of HIV transmission via vaginal and anal sex [50,51] to determine if breakage is a problem of epidemiologic concern.

Third, broader population surveys in Africa – not just among MSM and FSW – should assess lubricant use and accessibility, and whether African populations are actually using lubricants which are “condom compatible.” Further analysis of condom failure in Africa may test for correlations between breakage and non-compatible lubricant use.

Condom acceptance has come a long way since the Malawian student worried about *piri-piri* hot sauce seeping through holes in condoms. It is now time to shift focus to the *piri-piri* sauce itself – or actually the other “condiments” used for sexual lubrication – so we can better understand how these substances affect the African HIV epidemic.

## References

[1] Muraguri N, Temmerman M, Geibel S (2012). A decade of research involving men who have sex with men in sub-Saharan Africa: current knowledge and future directions. Sahara J.

[2] Beyrer C, Sullivan PS, Sanchez J, Dowdy D, Altman D, Trapence G (2012). A call to action for comprehensive HIV services for men who have sex with men. Lancet.

[3] Workowski KA, Berman S (2010). Centers for Disease Control and Prevention (CDC). Sexually transmitted diseases treatment guidelines, 2010. MMWR Recomm Rep.

[4] World Health Organization and UNAIDS (2001). The male latex condom: 10 condom programming fact sheets. http://www.unaids.org/en/media/unaids/contentassets/dataimport/publications/irc-pub01/jc003-malecondom-factsheets_en.pdf.

[5] International Organization for Standardization (2002). ISO 4074:2002 natural latex rubber condoms – requirements and test methods.

[6] ASTM International (2008). ASTM D3492 – 08 standard specification for rubber contraceptives (male condoms).

[7] ASTM International (2010). ASTM D7661 – 10 standard test method for determining compatibility of personal lubricants with natural rubber latex condoms.

[8] Department of Health and Human Services, Food and Drug Administration (2011). Food and drug administration modernization act of 1997: modifications to the list of recognized standards, recognition list number: 027. Fed Regist.

[9] Voeller B, Coulson AH, Bernstein GS, Nakamura RM (1989). Mineral oil lubricants cause rapid deterioration of latex condoms. Contraception.

[10] Pugh B, Englert M (1989). An evaluation of the effects of various lubricants on latex condoms.

[11] Rosen AD, Rosen T (1999). Study of condom integrity after brief exposure to over-the-counter vaginal preparations. South Med J.

[12] Grady WR, Tanfer K (1994). Condom breakage and slippage among men in the United States. Fam Plann Perspect.

[13] Trussell J, Warner DL, Hatcher RA (1992). Condom slippage and breakage rates. Fam Plann Perspect.

[14] Steiner M, Piedrahita C, Joanis C, Glover L, Spruyt A (1994). Condom breakage and slippage rates among study participants in eight countries. Int Fam Plan Perspect.

[15] Russell-Brown P, Piedrahita C, Foldesy R, Steiner M, Townsend J (1992). Comparison of condom breakage during human use with performance in laboratory testing. Contraception.

[16] Spruyt A, Steiner MJ, Joanis C, Glover LH, Piedrahita C, Alvarado G (1998). Identifying condom users at risk for breakage and slippage: findings from three international sites. Am J Public Health.

[17] de Wit JB, Sandfort TG, de Vroome EM, van Griensven GJ, Kok GJ (1993). The effectiveness of condom use among homosexual men. AIDS.

[18] Stone E, Heagerty P, Vittinghoff E, Douglas JM, Koblin BA, Mayer KH (1999). Correlates of condom failure in a sexually active cohort of men who have sex with men. J Acquir Immune Defic Syndr Hum Retrovirol.

[19] Gabbay M, Gibbs A (1996). Does additional lubrication reduce condom failure?. Contraception.

[20] Steiner M, Piedrahita C, Glover L, Joanis C, Spruyt A, Foldesy R (1994). The impact of lubricants on latex condoms during vaginal intercourse. Int J STD AIDS.

[21] Sparrow MJ, Lavill K (1994). Breakage and slippage of condoms in family planning clients. Contraception.

[22] European Working Group on HIV infection in Female Prostitutes (1993). HIV infection in European female sex workers: epidemiological link with use of petroleum-based lubricants. AIDS.

[23] Crosby R, Diclemente RJ, Yarber WL, Snow G, Troutman A (2008). An event-specific analysis of condom breakage among African American men at risk of HIV acquisition. Sex Transm Dis.

[24] Geibel S, Luchters S, King’ola N, Esu-Williams E, Rinyiru A, Tun W (2008). Factors associated with self-reported unprotected anal sex among male sex workers in Mombasa, Kenya. Sex Transm Dis.

[25] Management Sciences for Health (2012). Safety of medicines in sub-Saharan Africa: assessment of pharmacovigilance systems and their performance.

[26] Preston C, Valdez ML, Bond K (2012). Strengthening medical product regulation in low- and middle-income countries. PLoS Med.

[27] Department of Health and Human Services, Food and Drug Administration (2003). Over-the-counter drug products; safety and efficacy review. Fed Regist.

[28] Sonnenschein Nath & Rosenthal LLP (2004). Letter to dockets management branch (HFA-305), food and drug administration. http://www.fda.gov/ohrms/dockets/dailys/04/June04/062804/03n-0539-c000002-01-vol1.pdf.

[29] Personal Products Company (2004). Letter to dockets management branch (HFA-305), food and drug administration. http://www.fda.gov/ohrms/dockets/dailys/04/June04/062804/03n-0539-c000003-01-vol1.pdf.

[30] Cosmetic, Toiletry, and Fragrance Association (2004). Letter to dockets management branch (HFA-305), food and drug administration. http://www.fda.gov/ohrms/dockets/dailys/04/may04/051204/03n-0539-c00001-vol1.pdf.

[31] Department of Health and Human Services, Food and Drug Administration (2008). Status of certain additional over-the-counter drug category II active ingredients. Fed Regist.

[32] Fuchs EJ, Lee LA, Torbenson MS, Parsons TL, Bakshi RP, Guidos AM (2007). Hyperosmolar sexual lubricant causes epithelial damage in the distal colon: potential implication for HIV transmission. J Infect Dis.

[33] Sudol KM, Phillips DM (2004). Relative safety of sexual lubricants for rectal intercourse. Sex Transm Dis.

[34] Adriaens E, Remon JP (2008). Mucosal irritation potential of personal lubricants relates to product osmolality as detected by the slug mucosal irritation assay. Sex Transm Dis.

[35] Begay O, Jean-Pierre N, Abraham CJ, Chudolij A, Seidor S, Rodriguez A (2011). Identification of personal lubricants that can cause rectal epithelial cell damage and enhance HIV type 1 replication in vitro. AIDS Res Hum Retroviruses.

[36] Dezzutti CS, Brown ER, Moncla B, Russo J, Cost M, Wang L (2012). Is wetter better? An evaluation of over-the-counter personal lubricants for safety and anti-HIV-1 activity. PLoS One.

[37] Gorbach PM, Weiss RE, Fuchs E, Jeffries RA, Hezerah M, Brown S (2012). The slippery slope: lubricant use and rectal sexually transmitted infections: a newly identified risk. Sex Transm Dis.

[38] Braunstein S, van de Wijgert J (2005). Preferences and practices related to vaginal lubrication: implications for microbicide acceptability and clinical testing. J Womens Health (Larchmt).

[39] Onyango-Ouma W, Birungi H, Geibel S (2005). Understanding the HIV/STI risks and prevention needs of men who have sex with men in Nairobi, Kenya.

[40] Baral S, Burrell E, Scheibe A, Brown B, Beyrer C, Bekker LG (2011). HIV risk and associations of HIV infection among men who have sex with men in peri-urban Cape Town, South Africa. BMC Public Health.

[41] Lane T, Shade SB, McIntyre J, Morin SF (2008). Alcohol and sexual risk behavior among men who have sex with men in South African township communities. AIDS Behav.

[42] Poteat T, Diouf D, Drame FM, Ndaw M, Traore C, Dhaliwal M (2011). HIV risk among MSM in Senegal: a qualitative rapid assessment of the impact of enforcing laws that criminalize same sex practices. PLoS One.

[43] Fonck K, Kaul R, Kimani J, Keli F, MacDonald KS, Ronald AR (2000). A randomized, placebo-controlled trial of monthly azithromycin prophylaxis to prevent sexually transmitted infections and HIV-1 in Kenyan sex workers: study design and baseline findings. Int J STD AIDS.

[44] Gallo MF, Sharma A, Bukusi EA, Njoroge B, Nguti R, Jamieson DJ (2010). Intravaginal practices among female sex workers in Kibera, Kenya. Sex Transm Infect.

[45] Geibel S, Tun W, Tapsoba P, Kellerman S (2010). HIV vulnerability of men who have sex with men in developing countries: horizons studies, 2001–2008. Public Health Rep.

[46] The U.S. President’s Emergency Plan for AIDS Relief (PEPFAR) (2011). Technical guidance on combination HIV prevention. http://www.pepfar.gov/documents/organization/164010.pdf.

[47] WHO, FHI, UNFPA (2012). Use and procurement of additional lubricants for male and female condoms: WHO/UNFPA/FHI. Advisory note.

[48] Steiner MJ, Taylor D, Hylton-Kong T, Mehta N, Figueroa JP, Bourne D (2007). Decreased condom breakage and slippage rates after counseling men at a sexually transmitted infection clinic in Jamaica. Contraception.

[49] Leblanc MA, Pickett J, Dezzutti C, Fuchs E, Gorbach P, Fernandez-Romero J (2012). Are lubricants safe for rectal use? Next steps for researchers and advocates.

[50] Boily MC, Baggaley RF, Wang L, Masse B, White RG, Hayes RJ (2009). Heterosexual risk of HIV-1 infection per sexual act: systematic review and meta-analysis of observational studies. Lancet Infect Dis.

[51] Baggaley RF, White RG, Boily MC (2010). HIV transmission risk through anal intercourse: systematic review, meta-analysis and implications for HIV prevention. Int J Epidemiol.

